# Assessing the Usage of a Guideline-Driven Interactive Case Simulation Tool for Insomnia Screening and Treatment in an HIV Clinical Education Program

**Published:** 2013

**Authors:** Xuan Hung Le, Amneris E. Luque, Dongwen Wang

**Affiliations:** University of Rochester Medical Center, Rochester, NY, USA

**Keywords:** Online systems, interactive case simulation tool, system usage diagram, user-computer interface, practice guideline, medical education, clinical decision support systems, insomnia, HIV

## Abstract

Interactive case simulation tools (ICSTs) are important vehicles to disseminate medical knowledge. We conducted a study to examine the usage of an insomnia screening and treatment case simulation tool in an HIV clinical education program. Using system usage diagrams (SUDs) as an instrument, we quantified visit frequency and length of stay for different types of system resources. Preliminary results have shown that both recommendations and interactive decision diagrams were frequently used, with the former having a longer length of stay but fewer visits. Case simulation functions seemed to be able to engage users. Future research is required to verify the generalizability of the identified usage patterns, to investigate issues in usability design, and to perform correlation analyses on system usage and context parameters.

## Introduction

Online interactive systems are now widely employed for clinical practice and medical education [1–4]. Among the various types of such systems, an important category is used for decision assistance and case simulation on individual patients [1–2,5–6]. We name this type of system “interactive case simulation tools (ICSTs)”. ICSTs are frequently developed as a vehicle to disseminate medical knowledge. On the back-end, they are typically driven by a knowledge base. On the front-end, they support user interactions such as reviewing the process for patient management, examining the different options for clinical decisions, and entering case-specific data for individualized recommendations. In many systems, the original knowledge sources, for example, published clinical practice guidelines, are posted as electronic documents associated with these tools. These knowledge sources are considered as an essential system component to improve user acceptance.

There were a few efforts reported previously on development of ICSTs and their integration with back-end knowledge bases [5–6]. However, little research has been conducted on the actual use of these tools by the targeted audience for real applications. Understanding the usage pattern of such systems will provide important insights to improve their development, to facilitate integration with the back-end knowledge base, and to direct evaluation of their impacts upon knowledge dissemination. In this paper, we report the preliminary results from a study to assess the usage of a guideline-driven ICST for insomnia screening and treatment in an HIV clinical education program [7]. The findings from this study will: (1) examine the feasibility of a series of methods to perform usage analyses on ICSTs, (2) obtain initial measures on usage of specific system resources and user functions, (3) direct the next-stage analyses on usage patterns, and (4) provide preliminary data for future improvement of this tool to support the identified usage patterns and to address specific usability issues.

## Materials and Methods

### The HIV Clinical Education Program Hosting the ICSTs

The ICST of this study was developed as a category of online resources for the HIV Clinical Education Initiative (CEI). The CEI program is sponsored by the New York State (NYS) Department of Health (DOH) AIDS Institute (AI) to disseminate the latest HIV clinical knowledge to healthcare providers [8]. In addition to ICSTs, we have built learning modules, CME courses, and other training resources for the CEI online program. Currently, we have developed four ICSTs: (1) insomnia screening and treatment for HIV patients, (2) mental health screening for HIV patients, (3) HIV post-exposure prophylaxis (PEP) following occupational exposure in healthcare workers, and (4) HIV PEP following sexual assault. All these tools were based on the related clinical practice guidelines developed by the NYS DOH AI, with short versions published as quick reference guides for HIV primary care clinicians. For this study, we focused only on usage assessment of the insomnia screening and treatment tool.

### System Architecture and User Functions

The system architecture of the ICSTs reported in this study is based on an enhancement of the GLIF/GLEE framework [9–10]. It includes five system components: (1) a GLEE server hosting computer-interpretable clinical guidelines encoded in the GLIF format, (2) system interfaces to integrate the GLEE server with patient data and execution traces, (3) a set of GLEE clients, each corresponding to an application of a selected guideline to a specific patient case, (4) a Universal Presentation Layer (UPL) to integrate a GLEE client with the user interface of an application, and (5) user interface implementation and integration [6]. To build the front-end user functions for the insomnia screening and treatment tool, we developed a web-based system using JavaServer Pages at the server side and jQueryMobile at the client side. Although the user interface design targeted primarily mobile devices such as smartphones and tablets, this tool can be also accessed from desktops and laptops. For the version of this tool that has been integrated with the CEI website for public use, we simplified the back-end with hard-coded logic to improve system performance. To facilitate those healthcare providers only interested in ICSTs for particular clinical problems, we built four separate mobile apps for Android devices. These mobile apps are essentially a wrap-up of the corresponding web-based tools, and therefore provide the same set of user functions.

**Figure 1 -:**
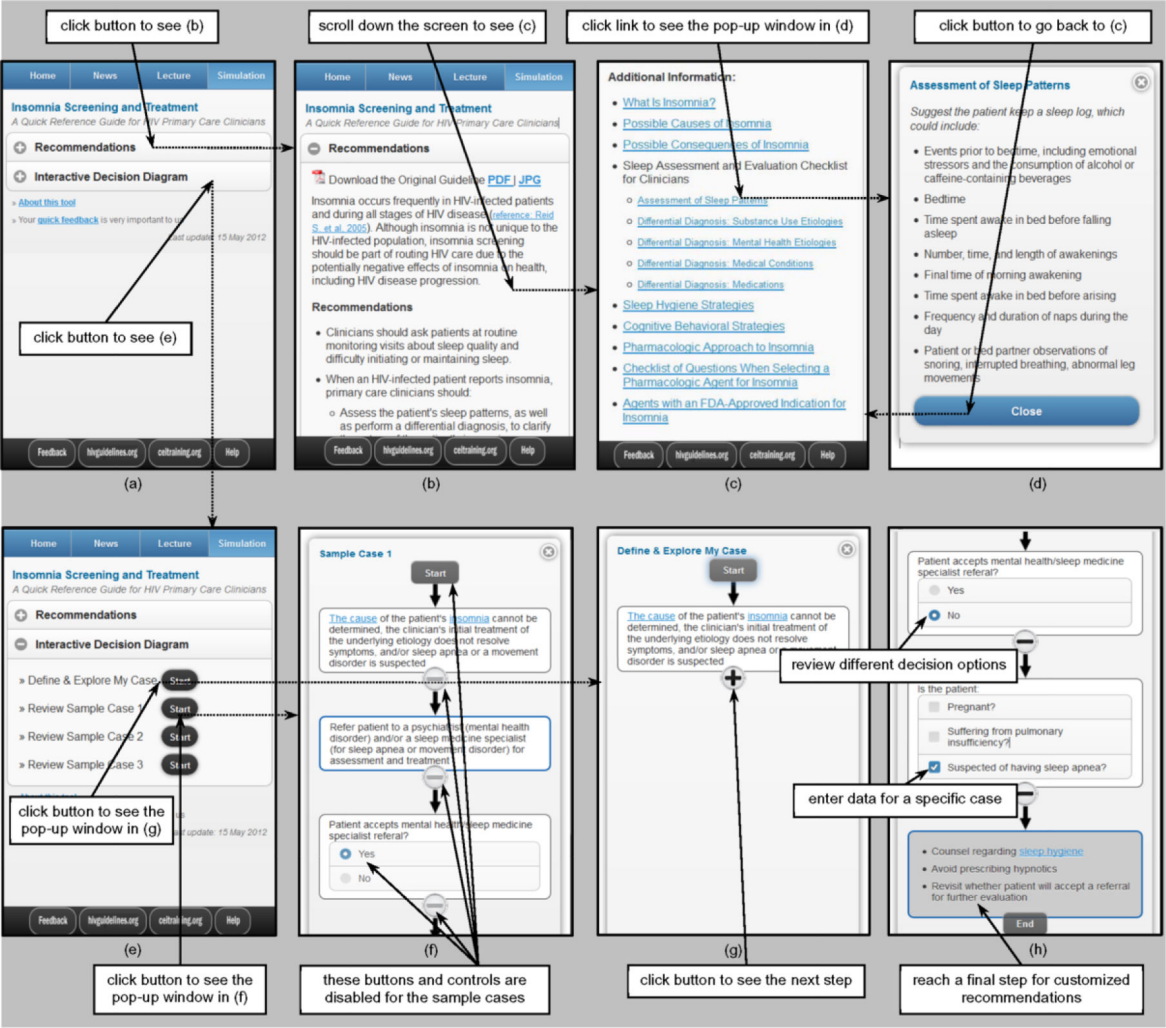
Selected Screenshots and Specific User Functions for the Insomnia Screening and Treatment Case Simulation Tool

For a specific ICST, we developed two sections of user functions. The first section is “*recommendations*”. This section is essentially a set of hyperlinked text pages transformed from the original quick reference guide. A clinician user can review the general recommendations, with options to follow the related links to check out the detailed information for specific clinical problems; he/she can also download the original quick reference guide in PDF or JPG format. The second section is “*interactive decision diagram*”. In this section, a clinician user can review the process of decision making on pre-defined sample cases; he/she can also explore his/her own patient cases through interactions with the system. Specifically, a user can check the decision process step by step, review the different decision options, and enter data for a particular patient case to examine the customized recommendations. At any point, a user can go to the related CEI or NYS HIV Clinical Guidelines website to obtain additional information. Selected screenshots and user functions for the insomnia screening and treatment case simulation tool are shown in Figure 1.

### Data Collection

To collect system usage data, we built tracking functions to monitor user actions on all screens of the system user interface. These user actions include: (1) opening a hyperlink to check the details of a recommendation, (2) clicking a button to go to the next step of the decision process, (3) selecting a specific decision option, and (4) entering the data for a particular patient case. We constructed specific types of system usage diagrams (SUDs) as instruments to record and analyze certain aspects of system usage. These SUDs are directed graphs with nodes and edges that can be assigned specific types of weights as system usage measures. In particular, we defined a node in SUD to represent a particular user action that could be launched from a specific screen of the system user interface; we created a directed edge in SUD to represent two consecutive user actions denoted by the nodes it spanned. We assigned a unique code for each user action (node). When a user action was tracked, we recorded its code as well as the timestamp. For the entire sequence of a user’s actions in a specific use session, we captured the associated session ID generated by the web server and the user’s IP address. We stored all these tracking data in a Microsoft SQL Server database. Specifically for this study, we queried the usage tracking database for all events recorded during the period from April 3, 2012 (when the insomnia screening and treatment tool was released to the public) to October 15, 2012 (when the tool was upgraded to a new version). To control the bias of data introduced by system testing, we excluded from analyses the usage by internal staff through sifting out the traffic coming from the IP addresses associated with their offices and homes.

### Usage Measure – Episodes of User Interactions

An episode of user interactions is a series of actions recorded during a short period of time – presumably for a specific purpose with a particular usage pattern. To define an episode of user interactions, we leveraged the two parameters recorded in the usage tracking database: session ID and IP address. It is important to note that using any one of these two parameters alone is insufficient to define a unique episode. For example, a clinician can use his/her office desktop to have multiple rounds of interactions with the tool on different days (multiple session IDs but single IP address); a clinician can also use his/her smartphone to access the tool at different locations (single session ID but multiple IP addresses). The combination of session ID and IP address, however, can serve the purpose well to define a unique episode of user interactions. Although this approach is still not perfect (for example, when a clinician uses his/her smartphone at a location where two mobile networks overlap, it is possible that the IP address could change in the middle of an episode), we believe it can effectively define episodes that are closest to the real-world use scenarios.

To reconstruct the episodes of user interactions, we queried the usage tracking database, categorized the user actions based on unique combinations of session ID and IP address, and sorted the user actions within each category (an episode) by their timestamps. When reviewing the recorded user actions, we have found that in rare situations it is necessary to leverage the timestamp associated with a user action to refine the definition of an episode. For example, a clinician could be interrupted in the middle of an episode or forgot to close the browser (or mobile app) after he/she finished a round of interactions. After a long time gap, he/she might return back to the tool to start another round of use. We believe in this case the new round of use after the long gap should be considered as a separate episode. Since these “long gaps” could occur in very different scenarios, it is impractical to define a single threshold for all applications. Therefore, we believe it would better to let individual investigators to handle this problem based on specific application contexts and patterns of user actions.

### Usage Measure – Visits to Specific System Resources

A major interest in analyzing the tool usage is to understand where the users go and how many users have visited specific system resources. To obtain this usage measure, we built a SUD, with each edge assigned a weight based on the accumulated frequency of user visits through that edge. When constructing this SUD, we enumerated the sequence of user actions in all episodes, mapped each two consecutive actions to a specific edge, and increased its weight by one each time an edge was processed. After completing weight calculation for all edges, we derived the weight of a node by adding up the weights of all its outgoing edges. This measure indicates the total number of visits to a specific system resource, which can be reached by performing the user action that node stands for.

### Usage Measure – Length of Stay on Specific Resources

Another major interest in analyzing the tool usage is to understand how long the users stay on specific system resources. To obtain this usage measure, we built another SUD, with each edge assigned a weight based on the average length of stay for all the visits corresponding to that edge. When constructing this SUD, we enumerated the sequence of user actions in all episodes, obtained the length of stay for each visit to a specific resource (the time interval between timestamps of two consecutive user actions), and calculated the weight for an edge. After completing weight calculation for all edges, we derived the weight of a node as the average length of stay for all its outgoing edges. This measure indicates the average length of stay on a specific system resource, which can be reached by performing the user action represented by that node.

### Data Analyses

To compare and to contrast the usage among the different parts of the insomnia screening and treatment tool, we segmented a SUD into three sub-graphs: (1) recommendation, which included only the nodes and edges under the “*recommendations*” section of the user functions, (2) sample-case, which included only the nodes and edges for the sample cases under the “*interactive decision diagram*” section, and (3) my-case, which included only the nodes and edges for user-defined cases under the “*interactive decision diagram*” section. To ensure completeness of the analyses, we created a fourth category, cross-over, which included the edges (and the associated nodes) spanning over two or more different sub-graphs. For each sub-graph, we calculated mean and standard deviation for visit frequency and length of stay. We performed all analyses using the SPSS statistical package.

## Results

### Episodes of User Interactions

We have recorded a total of 233 sessions, 209 IP addresses, and 239 unique combinations of session ID and IP address. Among these, we excluded from analyses 133 episodes in which a user opened the tool and immediately left (see discussion on this point later). To examine the problem of “long gaps” in the remaining episodes, we profiled the time intervals between two consecutive user actions for all sessions. As shown in Table 1, 512 out of the total 554 intervals (92%) were under 15 minutes. The remaining 42 intervals (8%) were all lengthier than 10 hours, with the longest gap recorded as 34 days. Therefore, it was obvious that we could use 10 hours as the threshold to define a “long gap”. After the 42 “long gaps” were processed (creating a new episode for the interactions after a long gap), we obtained a total of 148 episodes, from which we extracted the data of visit frequency and length of stay for further analyses. The number of user actions in these episodes scattered in a range from 1 to 33. In other words, the user performed 33 actions to interact with the system during the longest episode that has been recorded.

### Visits to Specific Resources

**Figure 2 -:**
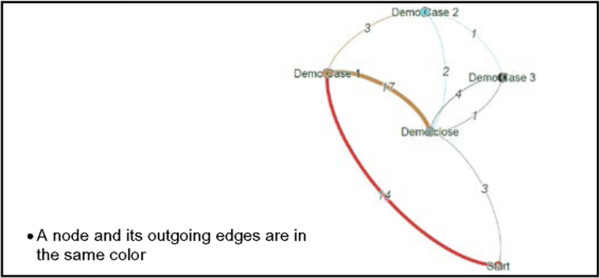
SUD on Visits (Sample-Case Sub-Graph)

**Figure 3 -:**
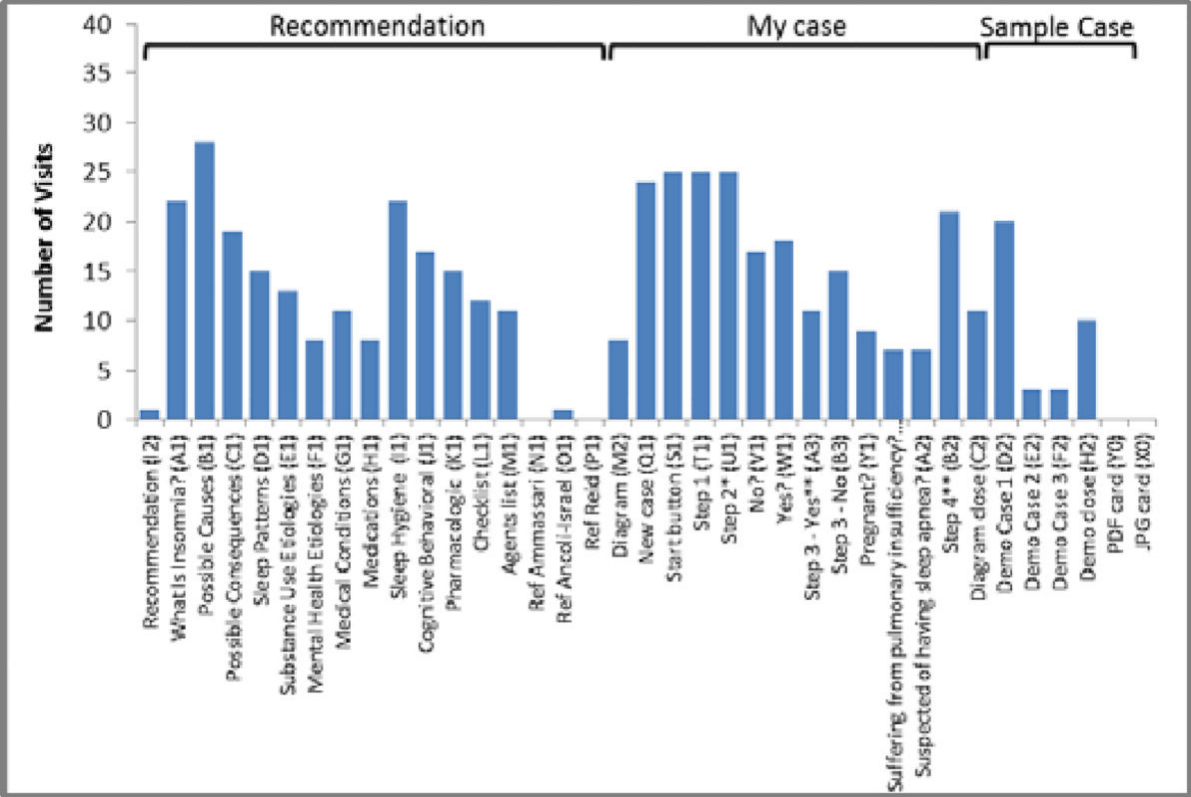
Visits to System Resources as Measured by Nodes

We have recorded a total of 512 visits to specific system resources in the insomnia screening and treatment tool. Among these, 202(39.5%) were in the “*recommendations*” section, 44(8.6%) were for the sample cases in the “*interactive decision diagram*” section, 223(43.5%) were for the user-defined cases in the “*interactive decision diagram*” section, and 43(8.4%) were cross-over visits spanning over two or more sub-graphs. The number of edges, the mean edge-weight, and the distribution of edge-weight for each sub-graph are shown in Table 2. The SUD on visits for the sample-case sub-graph is shown in Figure 2. Due to space limitation, we won’t be able to present the SUDs on visits for the other sub-graphs here, but they are available for review online [11]. With regard to user visits to specific nodes, their distributions are shown in Figure 3. Among the 37 unique system screens, 9 (3 in recommendation, 1 in sample-case, and 5 in my-case) recorded >=20 visits, 8 (4 in recommendation and 4 in sample-case) recorded <5 visits, and the remaining 20 (11 in recommendation, 1 in sample-case, and 8 in my-case) recorded 6–19 visits.

### Length of Stay on Specific Resources

**Figure 4 -:**
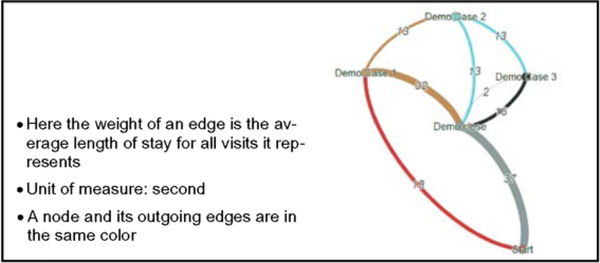
SUD on Length of Stay (Sample-Case Sub-Graph)

**Figure 5 -:**
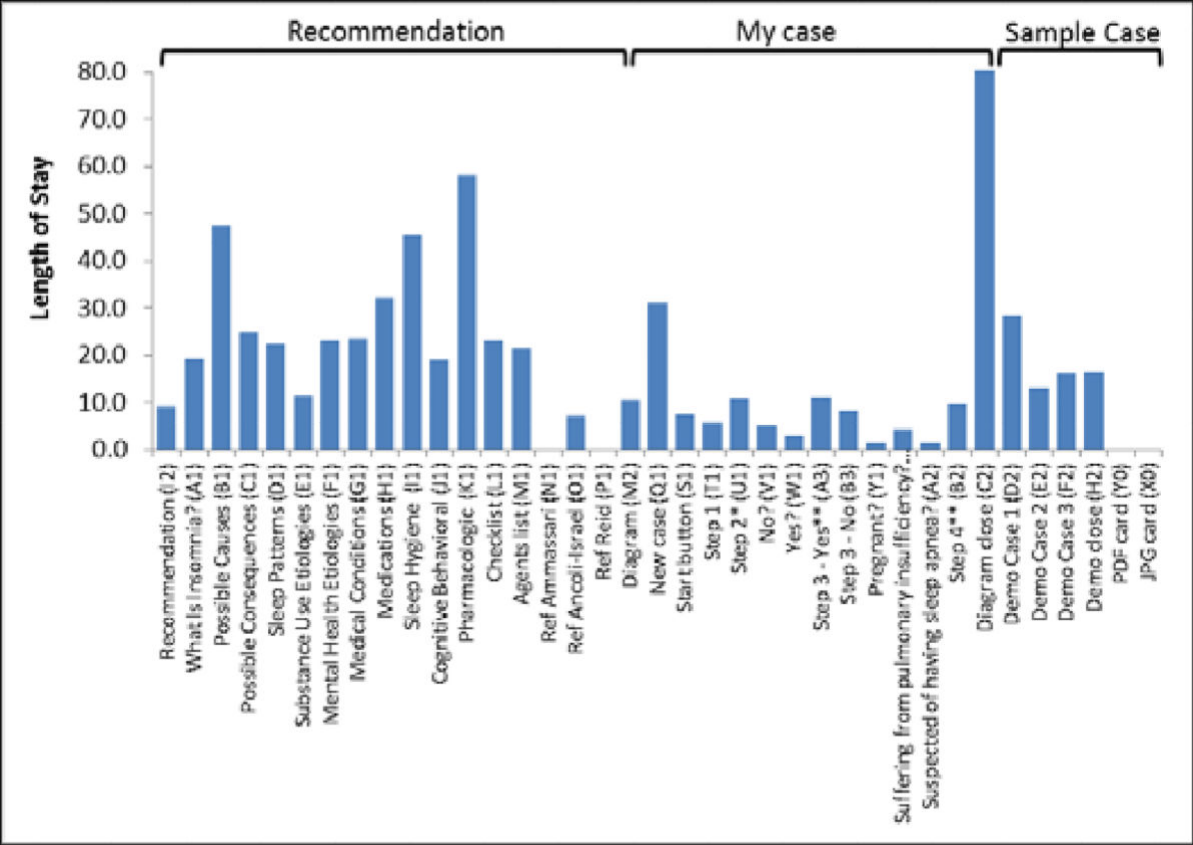
Length of Stay on Resources as Measured by Nodes

The average length of stay on a specific resource (system screen) was 34.86 seconds in the “*recommendations*” section, 21.91 seconds for the sample cases in the “*interactive decision diagram*” section, 18.73 seconds for the user-defined cases in the “*interactive decision diagram*” section, and 35.09 seconds for the cross-over visits. The distribution of edge-weight for each sub-graph is shown in Table 3. Results from the Kruskal-Wallis test have shown that the differences among the sub-graphs are statistically significant (p<0.001). Further analyses with Mann-Whitney test have shown significant differences between “*recommendations*” and “*my-cases*” (p<0.001). The SUD on length of stay for the sample-case sub-graph is shown in Figure 4. Additional SUDs are available for review online [11]. With regard to length of stay on specific nodes, distributions are shown in Figure 5. Consistent with the data in Table 3, the average length of stay on nodes in the “*recommendations*” section was significantly higher than those user-defined cases in the “*interactive decision diagram*” section.

## Discussion

In this study we explored a series of methods to assess the usage of the insomnia screening and treatment case simulation tool. In particular, we used the unique combinations of session ID and IP address to define an episode of user interactions. We profiled the time intervals between two consecutive user actions and identified the “long gap” problem. For this case study, we were able to make a relatively easy decision to resolve the “long gap” problem owing to the distribution of the time interval data (92% on the low-end, 8% on the high-end, none in the middle range). Generalizability of this pattern needs to be further examined in future studies.

An important methodology development from this study is to use network analyses to assess system usage. For this purpose, we defined SUDs for usage measures such as visit frequency and length of stay. We segmented a SUD into sub-graphs based on groups of system functions. In this way, we were able to compare and contrast system usage by different categories of user interactions. More important, there is a good chance that we can use other properties of the SUDs to explore additional aspects of system usage. For example, we can examine how well ICSTs engage users through analyses of the number of users leaving the system at specific locations of a SUD. This will be a direction of our research for the next step. With regard to the specific usage measures, the results from this study have shown that both the “*recommendations*” and “*interactive decision diagram*” sections were frequently used. In terms of visit frequency, the “*interactive decision diagram*” side recorded a higher usage than the “*recommendations*” side (see mean edge-weight in Table 2). In terms of length of stay, the situation was the opposite (see mean edge-weight in Table 3). The top three most popular topics in the “*recommendations*” section were: “*possible causes of insomnia*”, “*what is insomnia*”, and “*sleep hygiene strategies*”. This could be an objective reflection of the importance of these topics, or simply an artifact created by the order of the topic list (“*what is insomnia*” and “*possible causes of insomnia*” were the 1^st^ and 2^nd^ topics in the list, see Figure 1(c)). The three topics with the lengthiest user stay in the “*recommendations*” section were: “*pharmacologic approach to insomnia*”, “*possible causes of insomnia*”, and “*sleep hygiene strategies*”. This could be owing to the large volume of information and stronger user interests in these topics. The good number of visits but shorter length of stay for the “*interactive decision diagram*” section could be the consequence of better user engagement and the nature of the case simulation functions (easy user interaction and a small piece of new information after each click). Obviously, these preliminary findings from this case study need to be further examined in other applications.

From the usage data analyses, we have also found a few interesting phenomena worth further investigation. First, in a large number of sessions a user opened the tool and immediately left. Review of these cases has found that most of them happened after a user downloaded the mobile app and opened it to verify its successful installation. Therefore, excluding these episodes from usage analyses was an appropriate decision. Second, in a few scenarios of a user’s interactions with the tool, he/she opted to use the “back” button of the web browser or the Android device to return to a previous screen instead of the system function that has been built for the same purpose. For example, by clicking the “close” button on the screen shown in Figure 1(d), a user will close the pop-up window and return the system to the previous screen shown in Figure 1(c). Unfortunately, this built-in system function was under-used. Our conjecture was that the user wanted to continue to use the tool, but worried that clicking the “close” button would end the session and close the system. In future studies, we might want to investigate whether changing the wording from “close” to “back to previous page” will make a difference to user behaviors. Third, even after the “long gaps” were processed, the time intervals between two consecutive user actions could still be as long as 15 minutes (see Table 1). It is unknown whether this was just a normal use by a specific user or because of other reasons such as distraction of user during the use of the tool or usability issues. Follow-up user surveys/interviews may help clarify these questions. This is another direction to explore in the future.

In this initial usage assessment of the insomnia screening and treatment tool, we mainly focused on user actions (nodes), resources (edges), and system functions (sub-graphs). To further analyze usage patterns, we need to include other context parameters and examine their correlations with specific usage measures. These parameters include but are not limited to: (1) new users vs. returning users, (2) web-based system vs. native app, (3) small-screen devices (for example, smartphones) vs. large-screen equipment (for example, desktops and tablets), and (4) short episodes vs. long episodes. These correlation analyses are another direction for our future work.

## Conclusion

We have successfully performed an initial usage assessment on an insomnia screening and treatment case simulation tool. Through network analyses with SUDs, we have quantified the visit frequency and length of stay for different types of user functions. Results from these analyses have shown that both recommendations and interactive decision diagrams were frequently used, with the former having a lengthier user stay but smaller number of visits. The case simulation functions seemed to be able to engage users. Directions of future research include: (1) verifying whether the usage patterns identified from this case study can be generalized to other applications, (2) investigating certain issues in system usability design, and (3) performing correlation analyses on system usage and additional context parameters.

## Figures and Tables

**Table 1 -:** Time Intervals between Consecutive User Actions

Range of Time Intervals	Counts
1 second - 10 seconds	263
10 seconds - 1 minute	215
1 minute - 15 minutes	34
15 minutes - 1 hour	0
1 hour - 10 hours	0
10 hours - 20 hours	10
20 hours - 100 hours	26
100 hours - 1000 hours	6

**Table 2 -:** Visits to System Resources as Measured by Edges

Sections	1	2	3	4
Total Visits	202 (39.5%)	44 (8.6%)	223 (43.5%)	43 (8.4%)
Number of Edges	73 (46.8%)	8 (5.1%)	46 (29.5%)	29 (18.6%
Min Edge-Weight	1	1	1	1
Max Edge-Weight	17	17	25	7
Mean Edge-Weight	2.82	5.63	5.02	1.48
Std. Dev. Edge-Weight	003.10	6.23	8.60	1.18

* Sections: 1:recommendation, 2:sample-case, 3:my-case, 4:cross-over

**Table 3 -:** Length of Stay on Resources as Measured by Edges

Sections	1	2	3	4
Total Visits	202 (39.5%)	44 (8.6%)	223 (43.5%)	43 (08.4%)
Min Edge-Weight	2	1	1	1
Max Edge-Weight	601	139	815	500
Mean Edge-Weight	34.86	21.91	18.73	35.09
Std. Dev. Edge-Weight	63.36	23.73	85.76	79.66

* Sections: 1:recommendation, 2:sample-case, 3:my-case, 4:cross-over

** Unit of measure on length of stay: second

## References

[1] Holmberg C, Harttig U, Schultze MB, Boeing H. The potential of the Internet for health communication: the use of an interactive on-line tool for diabetes risk prediction. Patient Educ Couns. 2011 4;83(1):106–12. 20547029 10.1016/j.pec.2010.04.021

[2] Kassen EC, Williams RM, Kelly SP, Men’s use of an Internet-based decision aid for prostate cancer screening. J Health Commun. 2012;17(6):677–97. 21919646 10.1080/10810730.2011.579688

[3] Courteille O, Bergin R, Stockeld D, Ponzer S, Fors U. The use of a virtual patient case in an OSCE-based exam – a pilot study. Med Teach. 2008;30(3):e66–76. 18484444 10.1080/01421590801910216

[4] Brown R, Rasmussen R, Baldwin I, Wyeth P. Design and implementation of a virtual world training simulation of ICU first hour handover processes. Aust Crit Care. 2012 8;25(3):178–87. 22436543 10.1016/j.aucc.2012.02.005

[5] Peleg M, Shachak A, Wang D, Karnieli E. Using multi-perspective methodologies to study users’ interactions with the prototype front end of a guideline-based decision support system for diabetic foot care. Int J Med Inform. 2009;78:482–93. 19328739 10.1016/j.ijmedinf.2009.02.008

[6] Le XH, Luque A, Wang D. Development of guideline-driven mobile applications for clinical education and decision support with customization to individual patient cases. Proc AMIA Symp. 2012;1828.

[7] Insomnia Screening and Treatment Case Simulation. Available at: http://m.ceitraining.org/guidelines/insomnia. Accessed on Dec.7, 2012.

[8] New York State HIV Clinical Education Initiative. Available at: http://ceitraining.org/. Accessed on Dec.7, 2012.

[9] Boxwala AA, Peleg M, Tu S, GLIF3: a representation format for sharable computer-interpretable clinical practice guidelines. J Biomed Inform. 2004;37(3):147–61. 15196480 10.1016/j.jbi.2004.04.002

[10] Wang D, Peleg M, Tu S, Design and implementation of the GLIF3 guideline execution engine. J Biomed Inform. 2004;37(5):305–18. 15488745 10.1016/j.jbi.2004.06.002

[11] System usage data. Available at: http://birdlab.org/data/insomnia/graph.pdf. Accessed on Dec.7, 2012.

